# A Comparative Analysis of Nutritional Composition in *Acer truncatum* Leaves and Seeds Over the Growing Stages

**DOI:** 10.1002/fsn3.71252

**Published:** 2025-11-24

**Authors:** Xiangjun Ma, Rui Gao, Lei Gao, Xuexia Yuan, Tong Zhao, Haining Hao, Hongxia Du, Rongqi Zhai, Chan Zhang, Jingxiu Bi, Yutao Wang, Pingxiang Liu

**Affiliations:** ^1^ Laboratory of Quality and Safety Risk Assessment for Agro‐Products of the Ministry of Agriculture (Jinan), Institute of Quality Standard and Testing Technology for Agro‐Products Shandong Academy of Agricultural Sciences Jinan China

**Keywords:** *Acer truncatum*, fatty acids, nutritional quality evaluation, total flavonoids

## Abstract

*Acer truncatum* is a valuable source of bioactive compounds, yet the dynamics of these components throughout its growth cycle remain poorly characterized, limiting the optimization of harvest timing for maximal yield. This study quantified key phytochemicals in leaves and seeds across growth stages. In leaves, total flavonoids, chlorogenic acid, and gallic acid reached their highest levels in May (5.62% ± 0.1%, 3.38 ± 0.31, and 6.87 ± 0.14 mg/g, respectively). Quercetin was highest in June (1.80 ± 0.17 mg/g), whereas vitamin C and free amino acids peaked in September (38.73 ± 1.40 and 85.76 ± 0.56 mg/g, respectively) and moisture content peaked in April (74.34% ± 0.17%). For seeds, total flavonoids and quercetin content were highest on September 30 and September 15, respectively, whereas total amino acids and moisture peaked on August 31. No significant temporal variation was detected in chlorogenic acid, gallic acid, or kaempferol. Regarding fatty acids (FA) in seeds, total FA, unsaturated FA (UFA), and monounsaturated FA (MUFA) increased through Sep 30 (reaching 85.36 ± 1.67, 76.63 ± 1.65, and 50.83 ± 1.24 g/100 g, respectively) and then declined slightly. In contrast, polyunsaturated FA (PUFA) decreased to a minimum on September 30 (25.80 ± 0.90 g/100 g) before rising markedly by October 15 (40.60 ± 2.13 g/100 g), whereas saturated FA (SFA) remained stable. These findings demonstrate that the accumulation of characteristic components in *A. truncatum* is markedly influenced by growth stage. Multivariate analyses and integrated nutritional quality evaluation confirmed that May was optimal for leaves, whereas September 30 was the optimal harvest time for seeds. In a word, the present study not only enhances the database of characteristic components during various growth stages of *A. truncatum* , but also establishes a theoretical foundation for the development of diverse harvesting and processing technologies for *A. truncatum* .

## Graphical abstract

This study reveals that the accumulation of bioactive compounds in *Acer truncatum* leaves and seeds is highly dependent on growth stage. Multivariate analysis determined May as the optimal harvest time for leaves, and September 30 for seeds, when key phytochemicals and fatty acids were at their peaks. These findings provide a crucial basis for optimizing harvest timing to maximize yield and quality.

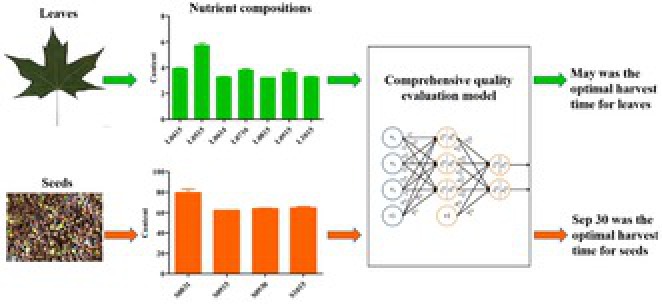

## 1 Introduction

*Acer truncatum* Bunge (Purpleblow maple, 2*n* = 2*x* = 26), a species native to northern and western China, belongs to the genus *Acer* and is endemic to China. Its samara resembles an ancient Chinese ingot, hence the Chinese name “yuan bao feng”.

Valued for its exceptional greenery and ornamental qualities, *A. truncatum* exhibits vigorous growth, a dense shading canopy, an elegant tree structure, and attractive leaf morphology. The young leaves are red, whereas the autumn foliage turns orange‐yellow or red, making it a premium species for autumn scenery (Fan et al. 2022).

In traditional practices within its native range, the leaves of *A. truncatum* were used to produce maple tea, and its roasted seeds were consumed directly. The hard, durable timber has been widely employed for making utensils and construction materials (Huang et al. 2007; Zhao et al. 2011). Furthermore, traditional medical texts document the efficacy of its roots and leaves in dispelling wind and dampness and relieving waist and back pain (Soyolt and Pei 2001).

In modern applications, *A. truncatum* has been developed into value‐added products such as instant tea and health tea (Fan et al. 2022).

Maple leaves are known for their pharmacological properties and are highly valued for their development. Specifically, the leaves of *A. truncatum* exhibit notable antitumor properties. Studies have demonstrated that its leaf extract significantly inhibits the proliferation of various human cancer cells, including liver (BEL‐7402), esophageal (CAES‐17), breast (MCF‐7), and gastric (BGC‐823) cancer lines. At a concentration of 60 μg/mL, the inhibition rates of these cell lines reached or exceeded 50%, indicating substantial anticancer potential (Zhao et al. 2006). Further contributing to its bioactivity, flavonoids from *A. truncatum* leaves play a significant role in antitumor activity. For instance, these compounds can inhibit the proliferation of Gejiu lung squamous cell strain (YTMLC) and mouse colon cancer CT‐26 cells, demonstrating time‐ and concentration‐dependent effects. Beyond antitumor effects, *A. truncatum* leaves also possess notable fatty acid synthase (FAS) inhibitory activity. Bioactive constituents containing gallic groups and flavonoid structures are considered the primary functional components responsible for this effect. Additionally, an abundant array of polyphenolic compounds in the leaves imparts notable antioxidant and antibacterial effects (Li et al. 2004; Zhang et al. 2008).

Owing to these diverse properties, *A. truncatum* leaves are regarded as a low‐cost and multifunctional source of bioactive compounds, demonstrating significant potential for applications in the functional food, pharmaceutical, and health product industries. However, the full exploitation of this potential is challenging. Studies have shown that the bioactive composition of *A. truncatum* leaves varies significantly throughout their growth cycle under the influence of environmental and climatic factors (Yeasmen and Orsat 2023). Yet, current research has been limited to functional nutrient analysis at only isolated time points, making it impossible to track the dynamic changes of characteristic components over time. This lack of temporal resolution hinders the identification of the optimal harvesting and extraction period, thereby constraining efficient utilization of the leaves.

In addition to the pharmacological properties of its leaves, the seeds of *A. truncatum* show significant potential in the treatment of neurological disorders, owing to their high content of nervonic acid (NA; C24:1Δ15, 24:1ω‐9, cis‐tetracos‐15‐enoic acid, ~5%) (Fan et al. 2018; Gu et al. 2019; Qiao et al. 2019). Moreover, in 2011, the seed oil, which is rich in unsaturated fatty acids (UFA, ~92%) and NA, was approved as a novel food ingredient by the Chinese Ministry of Health (http://www.nhc.gov.cn/). UFAs maintain membrane fluidity by reducing lipid packing density, thereby enabling cells to adapt to rigidifying conditions such as low temperature (DeMendoza and Pilon 2019). They are classified into two main categories: monounsaturated fatty acids (MUFAs), which contain one double bond in the carbon chain—with oleic acid (C18:1) being a representative example, and polyunsaturated fatty acids (PUFAs), which possess two or more double bonds, such as linoleic acid (LA, C18:2), linolenic acid (C18:3), and arachidonic acid (Liu, Shen, et al. 2023). Among them, PUFAs play vital roles in inflammatory responses, cancer prevention, cellular function, and lipid metabolism (Delmas and Aires 2025). For example, LA exhibits neuroprotective effects by suppressing the secretion of pro‐inflammatory cytokines such as TNF‐α and IL‐1β, thereby inhibiting microglial activation (Tan et al. 2022). Dietary DHA exerts anticancer effects by promoting ROS production to reduce tumor cell metastasis and invasion, as well as by inhibiting serine 437 phosphorylation on AKT through the PI3K‐AKT pathway to induce apoptosis (Yin et al. 2017). Furthermore, the specific biological functions of fatty acids are closely linked to their relative proportions and overall composition. During the maturation process of *A. truncatum* seeds, the fatty acid profile undergoes dynamic changes, influencing both functional efficacy and nutritional value. However, critical data gaps remain regarding which maturation stage yields seeds with the highest functional activity for neurological support or optimal nutritional quality. This lack of temporally specific profiling hinders the targeted development of *A. truncatum* seed oil as a high‐value functional product.

In this study, the contents of total flavonoids, chlorogenic acid, gallic acid, quercetin, kaempferol, VC, total 21 amino acids, and moisture in *A. truncatum* leaves collected in different months were determined. In addition to the aforementioned parameters, we also determined the fatty acid content in *A. truncatum* seeds during the maturation process. Finally, we conducted a comprehensive evaluation of the nutritional quality of leaves and seeds at different times using the evaluation model. On the basis of the findings of this study, the nutritional dynamics of *A. truncatum* leaves and seeds can be effectively characterized across developmental stages, providing critical insights into their quality variations. This knowledge facilitates the optimized utilization of *A. truncatum* resources and supports the sustainable growth of the woody industry in China.

## 2 Materials and Methods

### 2.1 Plant Material and Reagents

The plant specimens of *A. truncatum* were acquired from a dedicated cultivation base in Liaocheng City, China, for the purposes of this study. Leaf samples of *A. truncatum* were collected monthly in mid‐April through mid‐October 2023, yielding seven samples identified by their collection dates (MMDD) as L0415, L0515, L0614, L0716, L0815, L0915, and L1015. Samaras were collected on four dates (August 31, September 15, September 30, and October 15), and the corresponding samples were labeled S0831, S0915, S0930, and S1015. All collected samples were immediately placed in a portable cooler box for transport to the laboratory. The collected specimens were immediately frozen in liquid nitrogen for cryopreservation. A quarter portion of each sample was freeze‐dried. The resulting lyophilized material was ground into a powder using a mixer mill at 22,000 rpm, homogenized through a 60‐mesh sieve, and stored at −80°C pending further analysis. Throughout the sampling process, strict random sampling principles were adhered to. To ensure comprehensiveness and representativeness, samples were collected from various positions within each tree canopy, including the upper, middle, and lower layers, as well as from all four cardinal directions: east, south, west, and north.

The acetonitrile and methanol (High Performance Liquid Chromatography, HPLC grade) were purchased from Merck (Darmstadt, Germany). Deionized water was prepared by a Milli‐Q water purification system (Millipore, France). The formic acid was obtained from Rhawn (Shanghai, China). The Folin Ciocalteu reagent was purchased from Sangon Biotech (Shanghai, China). Chlorogenic acid, quercetin, kaempferol, and other reference standards were purchased from Source Leaf Biotechnology Co. Ltd. (Shanghai, China). Other reagents used in our work were brought from Sinopharm Chemical Reagent Co. Ltd. (Shanghai, China).

### 2.2 Ultrasound‐Assisted Extraction of Oil From *A. truncatum* Seeds

Twenty grams of *A. truncatum* seed powder was mixed with 400 mL of petroleum ether and extracted in a covered bottle using an ultrasonic device (250 W, 40 kHz, 40°C–60°C) for 45 min. Heat the mixture in an oven at 75°C overnight, and filter it using a Büchner funnel vacuum filtration apparatus with a 0.22 μm filter membrane. Preserve the filtrate. Remove the extraction solvent using a rotary evaporator (38°C, 85 rpm), and dry and concentrate the filtrate to a constant weight.

### 2.3 Determination of Several Nutritional Components

The total flavonoid content was analyzed according to NY/T 1295 and quantified using a calibration curve constructed with rutin as the standard. Chlorogenic acid, quercetin, gallic acid, kaempferol, and vitamin C (Vc) were determined using a modified HPLC with Ultraviolet Detection (HPLC‐UV) method on the basis of the procedure described by Yi et al. (2019). Free amino acids were analyzed by the ninhydrin colorimetric method using leucine as the standard, and their concentrations were expressed as mg per g DW. The moisture content of *A. truncatum* was determined by heating 10 g of chopped sample at 105°C for 6 h (Liu et al. 2024). Fatty acid composition in *A. truncatum* seed oil was analyzed according to the method specified in the China National Standards (GB 5009.168–2016) (Liang et al. 2019).

### 2.4 Data Processing and Statistical Analysis

All experiments were performed in triplicate, and results are expressed as mean ± standard deviation. Data were analyzed by one‐way ANOVA with Tukey's honestly significant difference (HSD) post hoc test for multiple comparisons using SPSS (SPSS Inc., Chicago, IL, USA). Differences were considered statistically significant at *p* < 0.05. Principal component analysis (PCA), hierarchical cluster analysis (HCA), and comprehensive quality evaluation modeling were conducted using SPSS. Orthogonal partial least squares‐discriminant analysis (OPLS‐DA) was performed using SIMCA software.

## 3 Results and Discussion

### 3.1 Temporal Dynamics of Eight Nutrient Compositions in *A. truncatum* Leaves From April to October

**FIGURE 1:**
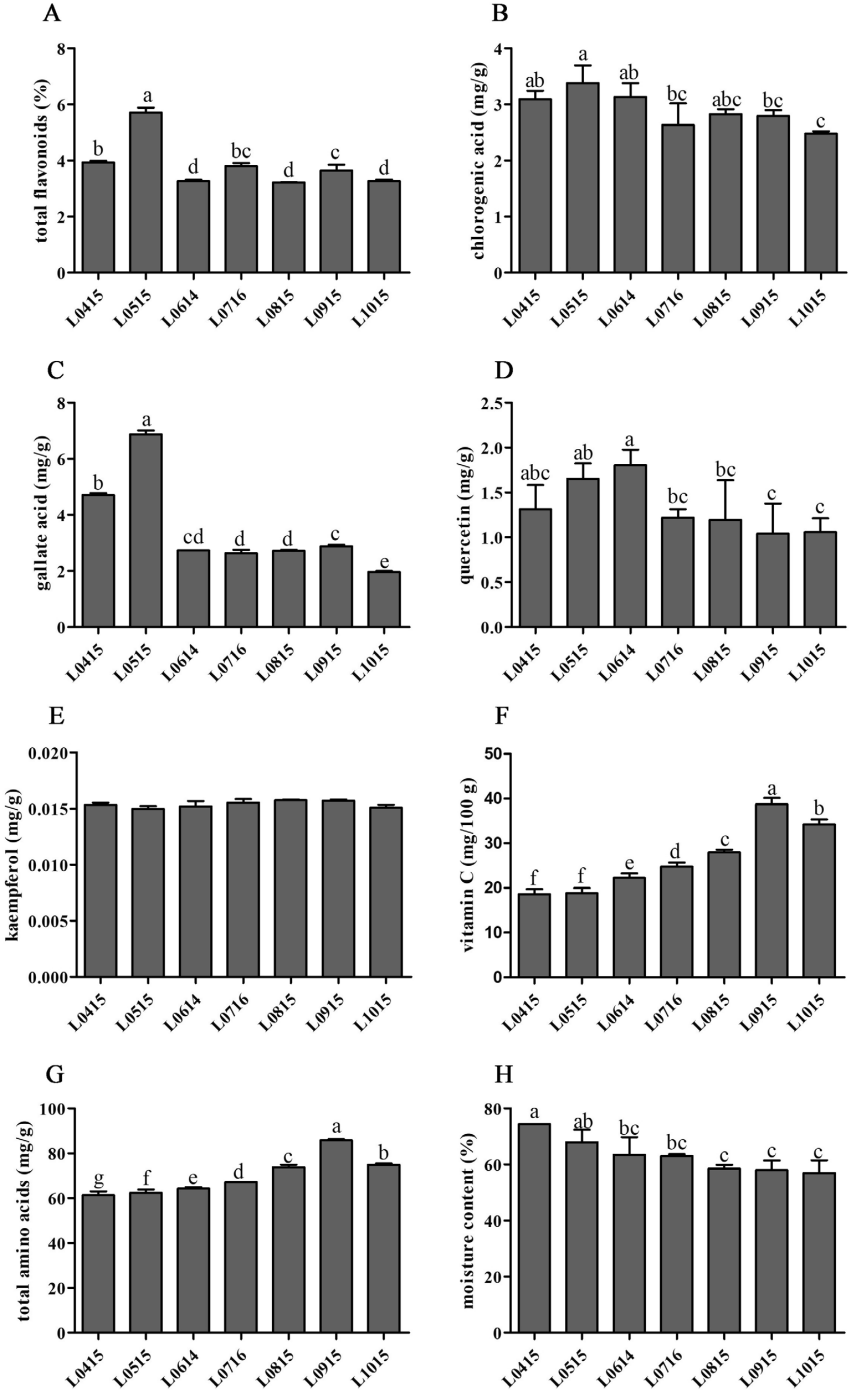
The levels of total flavonoids, chlorogenic, quercetin, gallic acid, kaempferol, VC, total 21 amino acids, and moisture content in different *A. truncatum* leaf samples. Significant differences are indicated by different letters (one‐way ANOVA with Tukey's test, *p* < 0.05) for multiple groups. Data are mean ± SD (*n* = 3).

As is well‐established, the nutritional composition of the same plant tissue can vary considerably across different growth stages (Zhou et al. 2024). According to Figure 1A, the total flavonoid content on May 15 was significantly higher than that in other months, reaching 5.62% ± 0.1%, and the total flavonoid content from June to October tended to be stable (3.55% ± 0.27%). This variation may be attributed to seasonal changes in environmental conditions and plant physiological priorities. During spring, the metabolic activity of newly emerged leaves is heightened, leading to substantial synthesis of total flavonoids as a protective mechanism against ultraviolet radiation and pathogens. In contrast, during summer, the metabolism of plant leaves tends to stabilize and resources are redirected toward growth, flowering, and fruiting activities, resulting in a decline in leaf flavonoid concentrations (Formato et al. 2022).

Chlorogenic acid, which is a plant secondary metabolite, has become a versatile natural food additive with diverse industrial applications because of its multifunctional activities, including antioxidant, anti‐inflammatory, antimicrobial, anticancer, antidiabetic, and anti‐obesity properties (He et al. 2025; Niggeweg et al. 2004). The chlorogenic acid content in maple leaves was high on April 15, May 15, and June 14, with the peak concentration (3.38 ± 0.31 mg/g) observed on May 15 (Figure 1B). This is because maple leaves enter a rapid growth stage in spring (April and May), and photosynthesis is enhanced, leading to active metabolic activities. Chlorogenic acid is an important secondary metabolite, and its synthesis increases as the leaf grows and metabolic activities become more active, thus reaching its peak in May. However, gallic acid (Figure 1C) exhibited a “single‐peak” pattern, where its content peaked on May 15 (labeled as “a”) and then continued to decline. This suggests that the growth period is a key factor regulating its accumulation, and the subsequent decrease in expression levels may be involved in oxidative stress or the conversion into other components (such as tannins) (Gessler et al. 2004; Rawat et al. 2018).

Quercetin (Figure 1D) showed significantly higher accumulation levels on June 14 (1.80 ± 0.17 mg/g), whereas kaempferol (Figure 1E) maintained relatively stable concentrations across all growth stages with no significant differences. Quercetin and kaempferol, as flavonol compounds, exhibit distinct seasonal accumulation patterns in maple leaves. The observed delayed accumulation pattern of quercetin likely stems from its biosynthetic pathway's dependence on multi‐step enzymatic reactions (e.g., catalysis by flavonol synthase) (Ma et al. 2003). This process requires precursor flavonoids (including total flavonoids, particularly dihydroflavonols) to accumulate to a critical threshold before efficient initiation (Zhu et al. 2024). In contrast, the stable levels of kaempferol throughout the growth period are likely due to *A. truncatum* 's fundamental physiological requirement (e.g., functioning as an antioxidant, controlling physiological processes, and strengthening defenses against pests and diseases), resulting in a dynamic equilibrium state between its synthesis and degradation (Belkheir et al. 2016).

In *A. truncatum* leaves, Vc and total amino acids—as two additional nutritional components—exhibited synchronized accumulation patterns during the late growth phase. As shown in Figure 1F, Vc content peaked on September 15 (labeled “a”, *p* < 0.05). Concurrently, total amino acids (Figure 1G) also reached a significant accumulation maximum on September (labeled “a”, *p* < 0.05). This coordinated accumulation aligns with metabolic characteristics during leaf maturation: as leaves develop, photosynthetic products (e.g., glucose) are increasingly channeled into Vc biosynthesis primarily through the L‐galactose pathway of ascorbate synthesis. Simultaneously, enhanced nitrogen assimilation capacity—evidenced by elevated glutamine synthetase (GS) activity—drives the expansion of the amino acid pool. The temporally synchronized accumulation of these compounds serves as a key indicator for determining the onset of the “nutritional maturity stage” in *A. truncatum* leaves (occurring around September 15).

Leaf moisture decreased monotonically from 74.34% ± 0.17% in April 15 to 56.99% ± 0.17% by October 15 (Figure 1H), reflecting natural desiccation during senescence. This trend is directly related to both leaf structural development (e.g., thickening of the cuticle, lignification of cells) and environmental adaptation (e.g., water redistribution following summer transpiration) (Ji et al. 2025).

In a word, these results clarify the dynamic accumulation patterns of various nutritional components in *A. truncatum* leaves, providing critical insights for their refined development and high‐value applications in the food industry. For instance, May is the optimal period for extracting total flavonoids and chlorogenic acid, making it suitable for developing functional foods or natural additives with antioxidant and anti‐inflammatory properties. June stands out for its high quercetin content, which can be targeted for the extraction of specific health‐promoting compounds. By September, Vc and amino acids simultaneously reach their peaks, indicating that the leaves have entered a nutritionally mature stage, ideal for processing into dietary supplements or sports food ingredients. These temporal characteristics offer clear practical guidance for the extraction of functional components, selection of high‐quality raw materials, and seasonal adaptive utilization of *A. truncatum* leaves.

### 3.2 Comprehensive Quality Assessment of *A. truncatum* Leaves in Different Growing Months

In addition to analyzing the main components of the leaves each month, we also need to evaluate their comprehensive nutritional value to fully leverage the potential of *A. truncatum* leaves. The comprehensive quality evaluation model, a mathematical approach developed from PCA, enables holistic multi‐indicator quality assessment while enhancing evaluation accuracy and efficiency (Liu, Wan, et al. 2023; Zhao et al. 2021). In this study, the original matrix (21 × 8) of quality indicators in *A. truncatum* leaves across different growth months was subjected to PCA. On the basis of the total eigenvalues (> 1) derived from the correlation coefficient matrix and the variance contribution rates of each principal component, principal components (P) were extracted. Ultimately, Principal Component 1 (P1), Principal Component 2 (P2), and Principal Component 3 (P3) were identified. As shown in Table 1, the eigenvalues corresponding to these principal components were 4.144, 1.957, and 1.127, respectively. P1 accounted for 57.334% of the total variance, representing over half of the information contained in the original variables. P2 contributed 27.073%, and P3 contributed 15.592%. The cumulative contribution rate of these three principal components reached 80.301%, indicating that they collectively represent 80.301% of the information from the measured quality indicators.

**TABLE 1:** Principal component analysis of *A. truncatum* leaves: Eigenvalues, variance explanation, and eigenvectors (components with eigenvalues > 1).

No	P1	P2	P3
X1: total flavonoids	0.343	0.452	0.087
X2: chlorogenic	0.136	0.126	−0.794
X3: quercetin	0.269	−0.382	0.205
X4: acid gallate	0.373	−0.039	0.134
X5: kaempferol flavonoids	−0.238	0.311	0.529
X6: Vc	−0.454	0.126	−0.134
X7: total 21 amino acids	−0.427	0.213	−0.057
X8: moisture	0.120	0.659	0.004
Eigenvalues	4.144	1.957	1.127
Proportion (%)	57.334	27.073	15.592
Cumulative (%)	46.040	67.781	80.301

The eigenvectors associated with the eigenvalues elucidate the relationships between the principal components and the original variables. The strength of these relationships is proportional to the absolute value of the eigenvector loadings. According to Table 1, P1 exhibited strong correlations with gallic acid, Vc, and total 21 amino acids. P2 was strongly correlated with total flavonoids, quercetin, and moisture. P3 showed strong correlations with chlorogenic acid and kaempferol flavonoids. To further explain the relationship between the quality indicators of *A. truncatum* leaves and the principal component factors, the functional expressions of the three principal components F1, F2, and F3, were obtained as follows, and X1–X8 represent the standardized variables of the quality indicators.

$$\begin{array}{cc} F1= & 0.343x1+0.136x2+0.269x3+0.373x4\\ & -0.238x5-0.454x6-0.427x7+0.120x8 \end{array}$$

$$\begin{array}{cc} F2= & 0.452x1+0.126x2-0.382x3-0.039x4\\ & +0.311x5+0.126x6+0.213x7+0.659x8 \end{array}$$

$$\begin{array}{cc} F3= & 0.087x1-0.794x2+0.205x3+0.134x4\\ & +0.529x5-0.134x6-0.057x7+0.004x8 \end{array}$$

As presented in Table 2, the membership values U1, U2, and U3 were calculated using the membership function *U* (*Xj*). Subsequent integration of these values with their corresponding weight values (*Wj*) yielded the comprehensive evaluation index (*D*‐value). The results indicate that the three leaf samples collected in May achieved the highest quality scores (0.867, 0.881, and 0.920), followed by the three April samples (0.634, 0.646, and 0.671). These findings suggest that young *A. truncatum* leaves harvested in spring, with peak quality observed in mid‐May, offer superior nutritional value and are highly suitable for product development. The relevant formulas are provided below.

$$U\;\left(\mathrm{Xj}\right)=\frac{\mathrm{Xj}-X\mathrm{mim}}{X\max-X\min},\;\left(j=1,2\ldots \ldots n\right)$$

$$\mathrm{Wj}=\mathrm{Pj}/\sum_{j=1\mathrm{pj}}^{n}$$

$$D=\sum_{j=1}^{n}\left[u\left(\mathrm{xj}\right)*\mathrm{wj}\right]$$

**TABLE 2:** Comprehensive evaluation of *A. truncatum* leaves' quality on the basis of the growth cycle.

No	F1	F2	F3	U1	U2	U3	*D*	Rank
L0415‐1	2.142	−1.743	−0.076	0.947	0.05	0.496	0.634	6
L0415‐2	1.882	−1.541	0.548	0.897	0.101	0.668	0.646	5
L0415‐3	2.188	−1.366	0.067	0.956	0.145	0.536	0.671	4
L0515‐1	1.872	1.795	0.415	0.896	0.942	0.632	0.867	3
L0515‐2	2.298	1.59	0.899	0.976	0.89	0.765	0.920	1
L0515‐3	2.423	2.024	−1.012	1	1	0.239	0.881	2
L0614‐1	0.681	−1.942	−0.02	0.67	0	0.512	0.464	13
L0614‐2	0.528	−1.263	0.513	0.641	0.171	0.659	0.516	9
L0614‐3	0.47	−1.768	0.464	0.63	0.044	0.645	0.474	12
L0716‐1	0.455	−0.006	−1.878	0.627	0.488	0	0.492	10
L0716‐2	−0.525	0.051	1.752	0.441	0.502	1	0.545	8
L0716‐3	−0.304	0.339	0.789	0.483	0.575	0.735	0.547	7
L0815‐1	−0.713	−0.591	−0.154	0.406	0.341	0.475	0.399	16
L0815‐2	−1.567	0.069	0.104	0.243	0.507	0.546	0.362	18
L0815‐3	−0.961	1.387	−0.614	0.358	0.839	0.348	0.487	11
L0915‐1	−2.851	1.333	0.504	0	0.826	0.656	0.326	20
L0915‐2	−1.878	1.522	0.64	0.184	0.873	0.694	0.450	14
L0915‐3	−1.929	1.77	−0.35	0.175	0.936	0.421	0.419	15
L1015‐1	−1.886	−1.177	0.662	0.183	0.193	0.7	0.266	21
L1015‐2	−1.081	−0.047	−0.321	0.336	0.478	0.429	0.389	17
L1015‐3	−1.243	−0.437	−0.026	0.305	0.379	0.51	0.357	19

### 3.3 Temporal Dynamics of Seven Nutrient Compositions in A. truncatum Seeds From August to October

**FIGURE 2:**
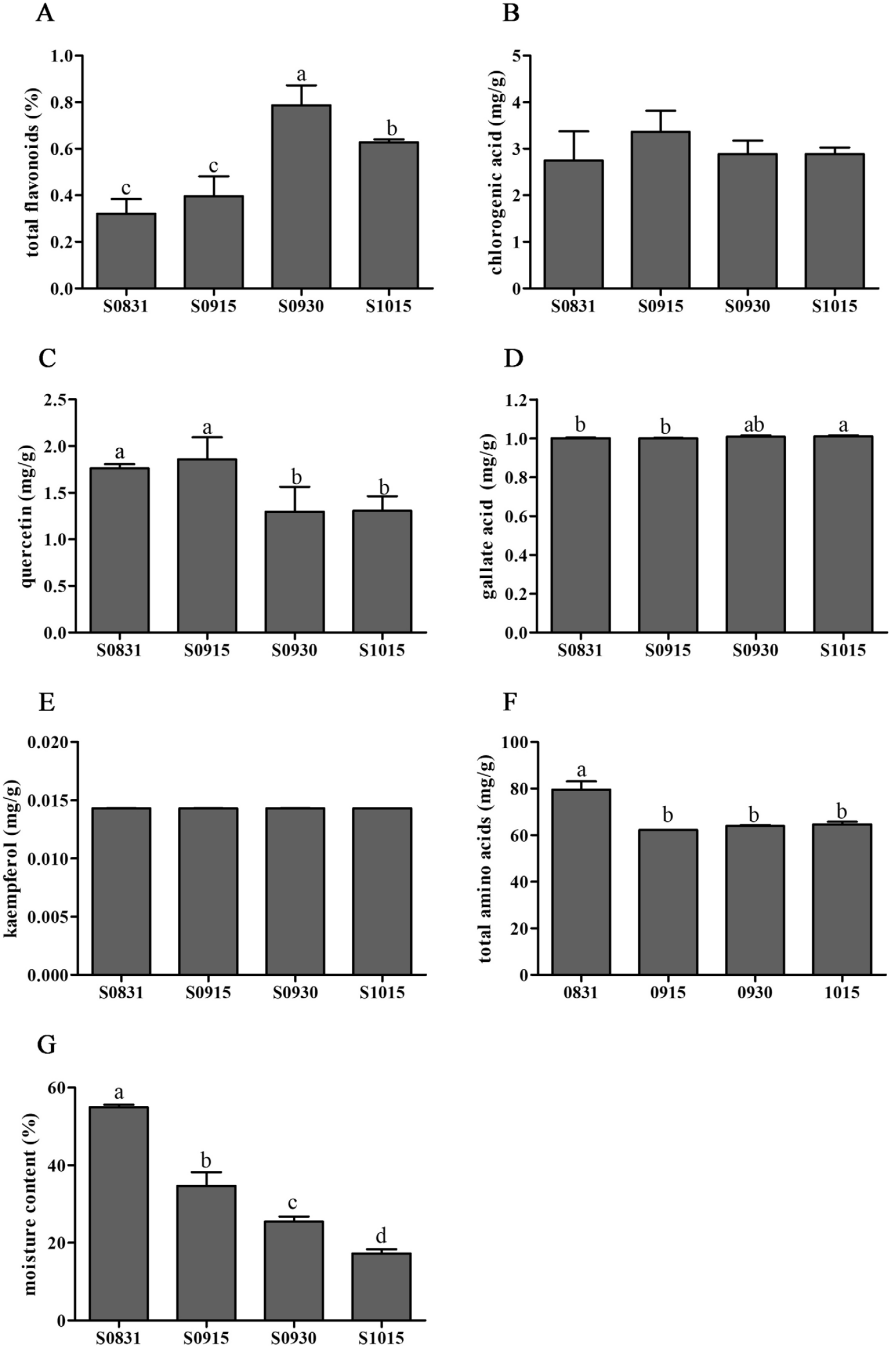
The levels of total flavonoids, chlorogenic, quercetin, gallic acid, kaempferol, total 21 amino acids, and moisture content in different *A. truncatum* seed samples. Significant differences are indicated by different letters (one‐way ANOVA with Tukey's test, *p* < 0.05) for multiple groups. Data are mean ± SD (*n* = 3).

The plant growth and development stages have a significant impact on the formation of nutrients in oil seeds (Sambanthamurthi et al. 2000). To characterize *A. truncatum* seed nutrient compositions, the contents of total flavonoids, chlorogenic acid, quercetin, gallic acid, kaempferol, total amino acids (21 types), and moisture were analyzed across different months (Figure 2). The results revealed that total flavonoid (Figure 2A) and quercetin (Figure 2C) contents peaked on September 30 and September 15, respectively. The highest levels of total amino acids (Figure 2F) and moisture (Figure 2G) occurred on August 31. Conversely, chlorogenic acid (Figure 2B), gallic acid (Figure 2D), and kaempferol (Figure 2E) levels showed no statistically significant variation across the sampling period, indicating their potential role as constitutive defense compounds in *A. truncatum* seeds. These compounds likely maintain basal stress resistance functions, including pathogen defense and UV protection (Farhadi et al. 2025). Given the elevated total flavonoid content detected in late September, seeds harvested during this period may possess enhanced antioxidant potential and skin‐brightening properties, as supported by previous studies (Tohidi et al. 2017).

### 3.4 The Dynamic Changes in the Content of Different Types of Fatty Acids in Seeds

**FIGURE 3:**
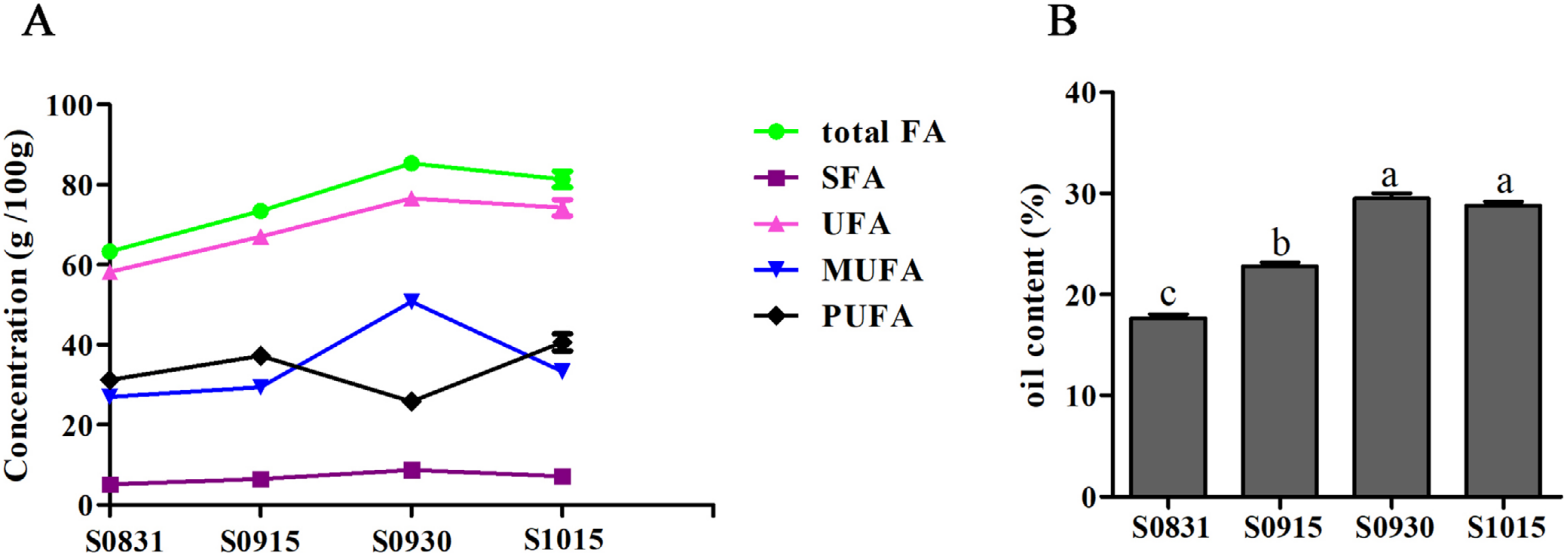
Total amount of SFA, UFA, MUFA, and PUFA during *A. truncatum* seed development (A), and (B) oil content at four sampling dates (S0831, S0915, S0930, and S1015). Significant differences are indicated by different letters (one‐way ANOVA with Tukey's test, *p* < 0.05) for multiple groups. Data are mean ± SD (*n* = 3).

During seed development of *A. truncatum* , the contents of total fatty acids (total FA), unsaturated fatty acids (UFA), and monounsaturated fatty acids (MUFA) increased significantly from August 31 to September 30, reaching peak values of 85.36 ± 1.67, 76.63 ± 1.65, and 50.83 ± 1.24 g/100 g, respectively, followed by a slight decline by October 15 (Figure 3A). In contrast, polyunsaturated fatty acids (PUFA) decreased sharply to their lowest level (25.80 ± 0.90 g/100 g) on September 30 before rising rapidly to 40.60 ± 2.13 g/100 g by mid‐October, whereas saturated fatty acids (SFA) remained stable throughout (Figure 3A). This pattern is consistent with other oil crops such as peanut and rapeseed, where total FA accumulation initially increases to a maximum and then slightly decreases during late maturation (Hills 2004; Liu et al. 2018; Zhang et al. 2023). The underlying reason may be that the vegetative organs of the plant begin to senesce at the later stages of development, leading to a rapid decline in photosynthetic capacity, whereas the synthesis of proteins and monosaccharides required for full maturity consumes part of the oil reserves (Baud et al. 2002; Hymowitz et al. 1972).

The nutritional quality and storability of edible oil are largely determined by the fatty acid composition of the oilseed (García‐González and Quintero‐Flórez 2023; Yildiz et al. 2025). MUFA (primarily oleic acid) is widely recognized for its positive effects on cardiovascular health and oxidative stability, making it a desirable component in edible oils (Gershuni 2018; Schwingshackl and Hoffmann 2014). Conversely, PUFA, despite being essential fatty acids, is more susceptible to oxidation, which can reduce oil shelf‐life and lead to rancidity (Okubanjo et al. 2019). SFA, whereas necessary for energy and biological functions, should be consumed in moderation to avoid potential cardiovascular risks (Gillingham et al. 2011). Therefore, a high MUFA content, combined with moderate SFA and low PUFA levels, is generally considered ideal for edible oils (Hooper et al. 2020). The *A. truncatum* oil seed composition observed on September 30—characterized by high oil content of 29.50% ± 0.527% (Figure 3B), elevated levels of UFA and MUFA, along with low PUFA content—achieves an optimal balance between nutritional quality and oxidative stability. In contrast, the sharp rise in PUFA by October 15 significantly increases susceptibility to oxidation, compromising long‐term storage stability. Furthermore, the oil yield on August 31 was significantly lower than that on September 30, reinforcing the latter's advantage not only in fatty acid profile but also in extraction efficiency. Thus, considering the dynamic changes in fatty acid composition and their direct impact on oil quality and storability, September 30 is established as the optimal harvest time for *A. truncatum* seeds, providing the best combination of nutritional value and functional stability.

### 3.5 Multivariate Statistical Analysis of 23 Free Fatty Acids in Seeds

**FIGURE 4:**
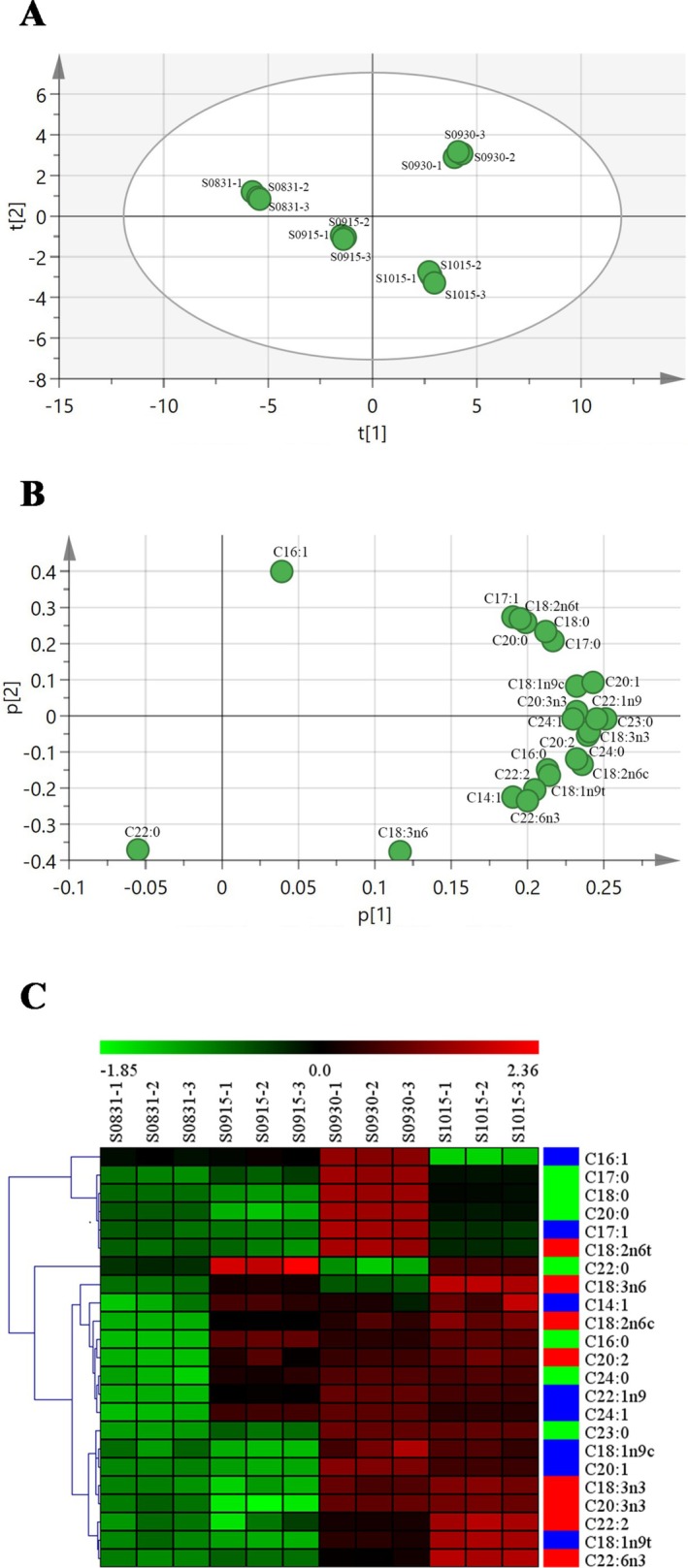
The score plot (A) and loading plot (B) of PCA and heatmaps (C) of four *A. truncatum* seed samples for about 23 fatty acids.

The PCA method has been widely used to evaluate the quality of agricultural products such as miscellaneous cassava tubers, kiwifruit, bayberry, and rice (Chen et al. 2024; Peng et al. 2023; Wei et al. 2017; Xu et al. 2022). By reducing the dimensionality of the data and eliminating overlapping information from numerous sources, the evaluation process is simplified, making it faster and more accurate than a single evaluation. In this study, PCA was used to analyze 23 free fatty acids in seeds to provide a comprehensive evaluation of nutritional quality differences in *A. truncatum* seeds across growth stages. As shown in Figure 4A, the 12 *A. truncatum* seed samples were divided into four regions on the basis of the first principal component (t [1]) and the second principal component (t [2]), which together accounted for nearly 80.8% of the total variance. In terms of sample distribution, the S0831 samples were primarily concentrated on the left side of the t [1] axis and within the 0–2 range of the t [2] axis. The S0915 and S1015 samples clustered in the −1 to −4 range of the t [2] axis, with a high degree of overlap between their distribution areas. In contrast, the S0930 samples were distinctly distributed on the right side of the t [1] axis and within the 2–4 range of the t [2] axis, showing clear separation from samples collected at the other three time points. This indicates that the S0930 samples have a significantly distinct nutritional composition compared to those from other months. The location of variables in the loading plot explains the reasons why certain observations form clusters in the score plot (Alkan et al. 2011). In the PCA loading plot (Figure 4B), components such as C17:1, C18:2n6, C20:0, C17:0, C18:0, and C20:1 exhibited positive and relatively high loading values. These components were primarily distributed on the right side of the p [1] axis, corresponding to the distribution area of the September 30 (S0930) samples in the score plot. This indicates that the contents of these components were higher in the *A. truncatum* seeds collected on September 30, and they are the key characteristic components responsible for the distinct separation of the samples from this time point from those of other stages.

HCA is another statistical method widely used in recent years. HCA classifies the research objects according to their degree of affinity in quality indicators, to analyze the similarity and difference between the nutritional quality of the samples (DeCarlo et al. 2019; Liu et al. 2024). Therefore, HCA (Figure 4C) was further conducted on the average contents of fatty acids in 12 *A. truncatum* seed samples. On the basis of their accumulation trends during the growth cycle, these fatty acids were clustered into three groups: Cluster 1 contained six fatty acids (including notable fatty acids such as C16:1, C17:0, C18:0, C20:0, C17:1 and C18:2n6t), whereas Cluster 2 (C18:3n6, C14:1, C18:2n6c, C16:0, C20:2, C24:0, C22:1n9 and C24:1) and Cluster 3 (C22:0, C23:0, C18:1n9c, C20:1, C18:3n3, C20:3n3, C22:2, C18:1n9t and C22:6n3) each contained eight. This demonstrates that fatty acids within the same group share similar accumulation patterns during growth.

**FIGURE 5:**
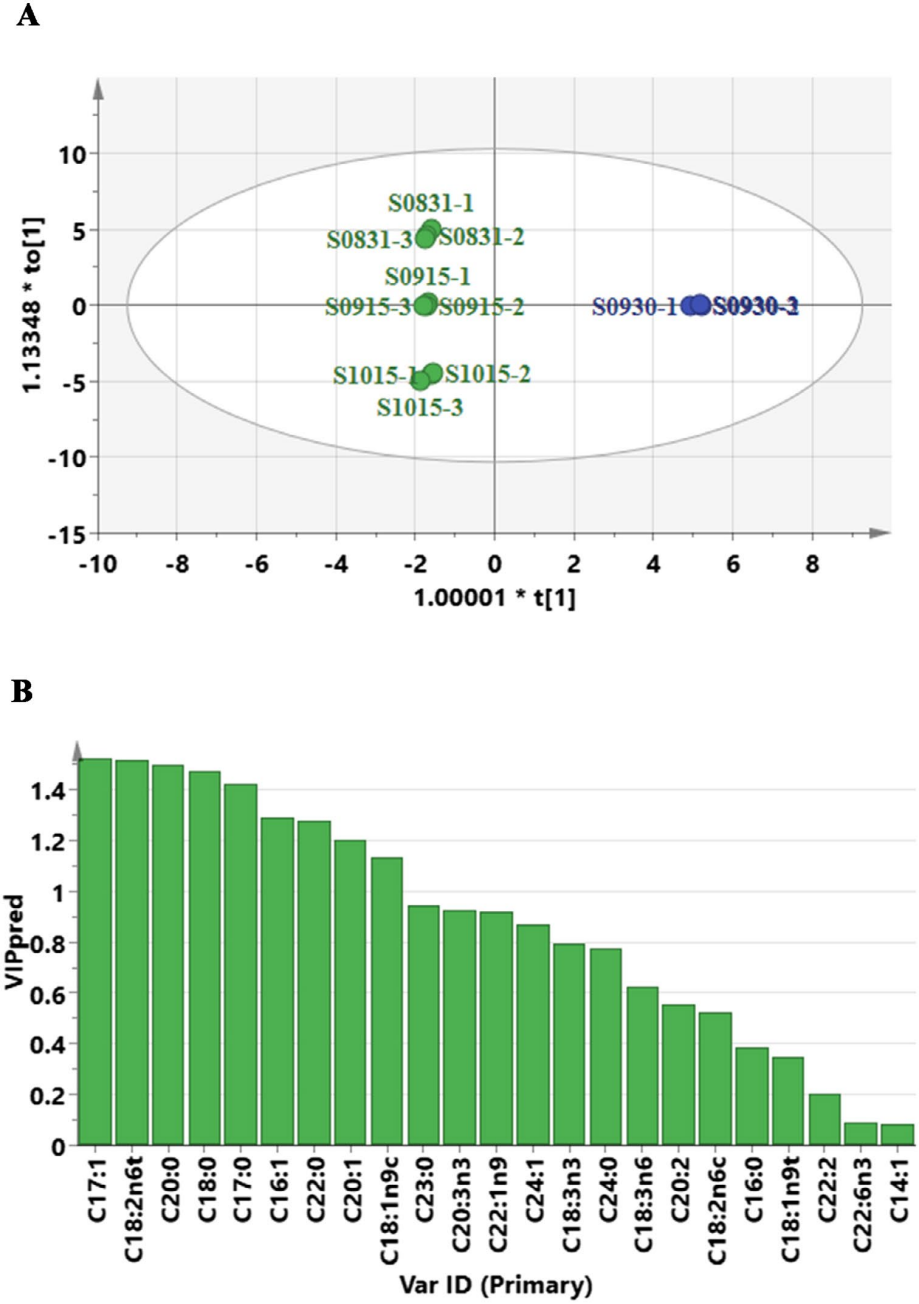
The OPLS‐DA score plot (A) and VIP plot (B) between the S0930 sample and other samples (S0831, S0915, and S1015).

To better identify the key fatty acids responsible for compositional differences in *A. truncatum* seed oil across developmental stages, an OPLS‐DA model was constructed on the basis of the four sample groups previously established by PCA and HCA. As a supervised method, OPLS‐DA effectively minimizes systemic noise and highlights variables that contribute most to group separation, offering improved classification efficiency over unsupervised PCA (Mais et al. 2018). The score plot of the OPLS‐DA model showed clear discrimination between the S0930 sample and samples from other time points (Figure 5A). The model exhibited high explanatory and predictive ability, with *R* ^2^ *Y* and *Q* ^2^ values of 0.925 and 0.998, respectively. On the basis of thresholds of VIP > 1 and fold change (FC) > 1, two groups of fatty acids were identified as significantly upregulated in S0930. The first group (VIP > 1 and FC > 1) included C17:1, C18:2n6t, C20:0, C18:0, C17:0, C16:1, C20:1, and C18:1n9c. A second group (FC > 1) consisted of C23:0, C20:3n3, C22:1n9, C24:1, C18:3n3, and C24:0 (Table 3). These results indicate that the S0930 oil possesses a functionally distinctive fatty acid profile. Notably, the high levels of nervonic acid (C24:1) suggest potential neuroprotective benefits (Yuan et al. 2023), whereas elevated oleic acid (C18:1n9c) may improve oxidative stability (Rodríguez‐Blázquez and Gómez‐Mejía 2023). The presence of essential fatty acids such as α‐linolenic acid (C18:3n3) further supports its nutritional value (Vinyard et al. 2025), collectively positioning S0930 *A. truncatum* seed oil as a promising functional ingredient.

**TABLE 3:** Variable importance in projection (VIP) and fold change (FC) of fatty acids in *A. truncatum* seed oil from multivariate models. The upward arrow (↑) indicates that the fatty acids content in sample S0930 is higher than in the other samples (S0831, S0915, and S1015). The asterisks denote significant differences based on Student’s t‐test (*P < 0.05, **P < 0.01, ***P < 0.001) for comparisons between S0930 and the other samples.

Var ID (primary) fatty acid name	VIP value	FC value
C17:1	1.524	1.985***↑
C18:2n6t	1.516	2.087***↑
C20:0	1.501	2.628***↑
C18:0	1.474	2.665***↑
C17:0	1.426	1.963***↑
C16:1	1.293	2.260***↑
C22:0	1.279	0.657
C20:1	1.203	1.251***↑
C18:1n9c	1.134	1.161**↑
C23:0	0.946	1.209*
C20:3n3	0.929	1.322*
C22:1n9	0.918	1.142*
C24:1	0.869	1.191*
C18:3n3	0.797	1.284*
C24:0	0.772	1.294*
C18:3n6	0.624	0.809
C20:2	0.554	1.138
C18:2n6c	0.521	1.099
C16:0	0.386	1.039
C18:1n9t	0.345	1.035
C22:2	0.203	0.980
C22:6n3	0.092	0.706
C14:1	0.084	0.983

### 3.6 Comprehensive Quality Assessment of *A. truncatum* Seeds in Different Growing Months

Xu et al. utilized a comprehensive quality evaluation model to calculate the comprehensive quality scores of different varieties' rapeseed oil, evaluated the quality of the harvested rapeseed oil, and selected the best variety (Xu et al. 2025). To quantitatively assess the quality of *A. truncatum* seed oil across maturation stages, we developed a multivariate model on the basis of monthly variations in fatty acids (23 types), SFA, UFA, MUFA, PUFA, bioactive compounds (total flavonoids, chlorogenic acid, quercetin, gallic acid, kaempferol), total amino acids, and moisture content. PCA was conducted on the correlation coefficient matrix, extracting components on the basis of eigenvalues exceeding 2 and variance contribution rates. Three principal components were identified: P1 (eigenvalue = 21.942, variance contribution = 64.537%), P2 (eigenvalue = 18.444, cumulative variance = 82.981%), and P3 (eigenvalue = 7.104, cumulative variance = 90.085%). Collectively explaining 90.085% of the total variance, these components effectively captured 90.085% of the original quality indicators' information (Table 4).

**TABLE 4:** Principal component analysis of *A. truncatum* seeds: Eigenvalues, variance explanation, and eigenvectors (components with eigenvalues > 2).

No	P1	P2	P3
X1: C16: 0	0.183	−0.139	0.237
X2: C17:0	0.181	0.201	0.100
X3: C18:0	0.176	0.226	−0.007
X4: C20: 0	0.164	0.252	−0.053
X5: C22: 0	−0.04	−0.358	0.247
X6: C23: 0	0.213	0.006	−0.032
X7: C24: 0	0.204	−0.043	0.160
X8: C14: 1	0.165	−0.207	0.129
X9: C16: 1	0.029	0.368	0.219
X10: C17: 1	0.158	0.265	0.046
X11: C18:1n9t	0.182	−0.142	−0.230
X12: C18:1n9c	0.196	0.095	−0.143
X13: C18:2n6t	0.161	0.258	0.034
X14: C18:2n6c	0.202	−0.119	0.048
X15: C18:3n6	0.104	−0.344	−0.077
X16: C20: 1	0.204	0.101	−0.082
X17: C18:3n3	0.202	−0.026	−0.167
X18: C20: 2	0.198	−0.104	0.146
X19: C22: 1n9	0.208	−0.002	0.134
X20: C20: 3n3	0.195	0.028	−0.242
X21: C22: 2	0.174	−0.177	−0.189
X22: C24: 1	0.196	−0.002	0.250
X23: C22: 6n3	0.171	−0.206	−0.174
X24: SFA	0.195	0.157	0.057
X25: UFA	0.212	−0.033	0.016
X26: MUFA	0.211	0.043	0.005
X27: PUFA	0.199	−0.137	0.035
X28: total flavonoids	0.166	0.095	−0.068
X29: chlorogenic acid	0.017	−0.096	0.375
X30: quercetin	−0.046	−0.147	−0.002
X31: gallic acid	0.149	−0.038	−0.295
X32: kaempferol	−0.007	0.06	0.255
X33: total amino acids	−0.166	0.095	−0.353
X34: moisture	−0.193	0.151	0.080
Eigenvalues	21.942	6.271	2.415
Proportion (%)	64.537	18.444	7.104
Cumulative (%)	64.537	82.981	90.085

To further elucidate relationships between *A. truncatum* seed quality indicators and principal component factors, we derived three principal component expressions (F1, F2, and F3) from characteristic vectors associated with 23 fatty acids, SFA, UFA, MUFA, PUFA, total flavonoids, chlorogenic acid, quercetin, gallic acid, kaempferol, total amino acids, and moisture content. Nutritional indicator concentrations were standardized to generate new variables (denoted X1‐X34) for this analysis. The comprehensive evaluation value D was obtained using the aforementioned method, as shown in Table 5. On the basis of the ranking of the comprehensive evaluation indicators, the top 3 samples were S0930‐1, S0930‐2, and S0930‐3, whereas the sample with the poorest ranking was S0831‐1, S0831‐2, and S0831‐3. This result indicates that the nutritional value of the *A. truncatum* seed on September 30 is the highest, so the collection of *A. truncatum* seeds should be carried out at this time point.

$$\begin{array}{cc} F1= & 0.858x1+0.847x2+0.823x3+0.767x4\\ & -0.189x5+0.996x6+0.955x7+0.775x8\\ & +0.135x9+0.738x10+0.851x11+0.919x12\\ & +0.755x13+0.946x14+0.485x15+0.955x16\\ & +0.948x17+0.929x18+0.976x19+0.912x20\\ & +0.815x21+0.918x22+0.803x23+0.914x24\\ & +0.994x25+0.99x26+0.934x27+0.777x28\\ & +0.08x29-0.214x30+0.7x31-0.034x32\\ & -0.776x33-0.903x34 \end{array}$$

$$\begin{array}{cc} F2= & -0.348x1+0.503x2+0.565x3+0.631x4\\ & -0.897x5+0.014x6-0.108x7-0.519x8\\ & +0.921x9+0.663x10-0.355x11+0.237x12\\ & +0.647x13-0.297x14-0.861x15+0.252x16\\ & -0.064x17-0.261x18-0.006x19+0.071x20\\ & -0.444x21-0.006x22-0.517x23+0.392x24\\ & -0.083x25+0.107x26-0.342x27+0.238x28\\ & -0.24x29-0.369x30-0.094x31+0.149x32\\ & +0.238x33+0.377x34 \end{array}$$

$$\begin{array}{cc} F3= & 0.368x1+0.155x2-0.011x3-0.082x4\\ & +0.384x5-0.05x6+0.249x7+0.2x8\\ & +0.341x9+0.072x10-0.358x11-0.222x12\\ & +0.053x13+0.075x14-0.119x15-0.128x16\\ & -0.26x17+0.227x18+0.209x19-0.376x20\\ & -0.293x21+0.388x22-0.27x23+0.088x24\\ & +0.025x25+0.007x26+0.054x27-0.106x28\\ & +0.582x29-0.003x30-0.458x31+0.397x32\\ & -0.549x33+0.124x34 \end{array}$$

**TABLE 5:** Comprehensive evaluation of *A. truncatum* seed quality on the basis of the growth cycle.

No	F1	F2	F3	U1	U2	U3	*D*	Rank
S0831‐1	−1.67	0.216	−0.99	0	0.468	0	0.089	11
S0831‐2	−1.651	0.083	−0.934	0.006	0.413	0.035	0.085	12
S0831‐3	−1.57	−0.006	−0.911	0.033	0.376	0.049	0.097	10
S0915‐1	−0.222	−0.917	0.48	0.473	0	0.904	0.380	8
S0915‐2	−0.257	−0.879	0.431	0.462	0.016	0.874	0.373	9
S0915‐3	−0.224	−0.913	0.515	0.473	0.002	0.926	0.381	7
S0930‐1	1.367	1.317	0.021	0.993	0.922	0.622	0.879	2
S0930‐2	1.388	1.494	−0.111	1	0.995	0.541	0.891	1
S0930‐3	1.271	1.506	−0.114	0.962	1	0.539	0.867	3
S1015‐1	0.483	−0.592	0.514	0.704	0.134	0.925	0.560	5
S1015‐2	0.441	−0.512	0.463	0.69	0.167	0.894	0.555	6
S1015‐3	0.642	−0.797	0.636	0.756	0.049	1	0.584	4

## 4 Conclusions

This study investigates the dynamic changes in key bioactive compounds in the leaves and seeds of *A. truncatum* across different growth stages. The findings reveal that the accumulation of these phytochemicals is highly dependent on the plant's developmental phase. In leaves, the optimal harvest time was determined to be May 15, as it corresponded with peak levels of total flavonoids, chlorogenic acid, and gallic acid. For seeds, the optimal harvest date was September 30, characterized by the highest content of total flavonoids, total FA, UFA, and MUFA, despite a temporary dip in PUFA on that date, which subsequently increased. Multivariate analysis confirmed these time points as ideal for maximizing the yield of valuable components. The research provides crucial data on the temporal variation of characteristic compounds in *A. truncatum* , establishing a theoretical basis for optimizing harvest timing and developing targeted processing technologies to enhance the utilization of this bioactive‐rich plant.

## Author Contributions

**Xiangjun Ma:** investigation, visualization, and writing – original draft. **Rui Gao:** investigation. **Lei Gao:** writing – review and editing. **Xuexia Yuan:** writing – review and editing. **Tong Zhao:** conceptualization. **Haining Hao:** formal analysis. **Hongxia Du:** methodology. **Rongqi Zhai:** investigation. **Chan Zhang:** investigation, methodology. **Jingxiu Bi:** investigation, methodology, formal analysis, visualization, writing – original draft, writing – review and editing. **Yutao Wang:** funding acquisition and writing – review and editing. **Pingxiang Liu:** funding acquisition and writing – review and editing.

## Conflicts of Interest

The authors declare no conflicts of interest.

## Data Availability

The data that support the findings of this study are available from the corresponding author upon reasonable request.

## References

[fsn371252-bib-0001] Alkan, D. , F. Tokatli , and B. Ozen . 2011. “Phenolic Characterization and Geographical Classification of Commercial Extra Virgin Olive Oils Produced in Turkey.” Journal of the American Oil Chemists' Society 89: 261–268. 10.1007/s11746-011-1917-6.

[fsn371252-bib-0002] Baud, S. , J. P. Boutin , M. Miquel , L. Lepiniec , and C. Rochat . 2002. “An Integrated Overview of Seed Development in *Arabidopsis thaliana* Ecotype WS.” Plant Physiology and Biochemistry 40: 151–160. 10.1016/S0981-9428(01)01350-X.

[fsn371252-bib-0003] Belkheir, A. K. , M. Gaid , B. Liu , R. Hänsch , and L. Beerhues . 2016. “Benzophenone Synthase and Chalcone Synthase Accumulate in the Mesophyll of *Hypericum perforatum* Leaves at Different Developmental Stages.” Frontiers in Plant Science 7: 921. 10.3389/fpls.2016.00921. 27446151 PMC4926534

[fsn371252-bib-0004] Chen, L. , R. Chen , and E. M. Atwa . 2024. “Nutritional Quality Assessment of Miscellaneous Cassava Tubers Using Principal Component Analysis and Cluster Analysis.” Food 13: 1861. 10.3390/foods13121861. PMC11203269 38928804

[fsn371252-bib-0005] DeCarlo, A. , S. Johnson , K. I. Okeke‐Agulu , et al. 2019. “Compositional Analysis of the Essential Oil of *Boswellia dalzielii* Frankincense From West Africa Reveals Two Major Chemotypes.” Phytochemistry 164: 24–32. 10.1016/j.phytochem.2019.04.015. 31071599

[fsn371252-bib-0006] Delmas, D. , and V. Aires . 2025. “Polyunsaturated Fatty Acids: New Molecular Mechanisms and Nutritional Therapeutic Challenges.” Nutrients 17: 588. 39940446 10.3390/nu17030588 PMC11820617

[fsn371252-bib-0007] DeMendoza, D. , and M. Pilon . 2019. “Control of Membrane Lipid Homeostasis by Lipid‐Bilayer Associated Sensors: A Mechanism Conserved From Bacteria to Humans.” Progress in Lipid Research 76: 100996. 10.1016/j.plipres.2019.100996. 31449824

[fsn371252-bib-0008] Fan, H. , L. Sun , L. Yang , et al. 2018. “Assessment of the Bioactive Phenolic Composition of *Acer truncatum* Seed Coat as a Byproduct of Seed Oil.” Industrial Crops and Products 118: 11–19. 10.1016/j.indcrop.2018.03.030.

[fsn371252-bib-0009] Fan, Y. , F. Lin , R. Zhang , M. Wang , R. Gu , and C. Long . 2022. “ *Acer truncatum* Bunge: A Comprehensive Review on Ethnobotany, Phytochemistry and Pharmacology.” Journal of Ethnopharmacology 282: 114572. 10.1016/j.jep.2021.114572. 34487848

[fsn371252-bib-0010] Farhadi, N. , M. Moghaddam , S. Farsaraei , K. Babaei , and A. G. Pirbalouti . 2025. “Phytochemical Profiling and Antioxidant Capacity of *Achillea santolina* at Different Phenological Stages.” Scientific Reports 15: 25885. 10.1038/s41598-025-11873-3. 40670593 PMC12267485

[fsn371252-bib-0011] Formato, M. , F. Scharenberg , S. Pacifico , and C. Zidorn . 2022. “Seasonal Variations in Phenolic Natural Products in *Fagus sylvatica* (European Beech) Leaves.” Phytochemistry 203: 113385. 10.1016/j.phytochem.2022.113385. 35998829

[fsn371252-bib-0012] García‐González, A. , and A. Quintero‐Flórez . 2023. “Virgin Olive Oil Ranks First in a New Nutritional Quality Score due to Its Compositional Profile.” Nutrients 15: 2127. 10.3390/nu15092127. 37432257 PMC10180740

[fsn371252-bib-0013] Gershuni, V. M. 2018. “Saturated Fat: Part of a Healthy Diet.” Current Nutrition Reports 7: 85–96. 10.1007/s13668-018-0238-x. 30084105

[fsn371252-bib-0014] Gessler, A. , C. Keitel , M. Nahm , and H. Rennenberg . 2004. “Water Shortage Affects the Water and Nitrogen Balance in Central European Beech Forests.” Plant Biology (Stuttgart, Germany) 6: 289–298. 10.1055/s-2004-820878. 15143437

[fsn371252-bib-0015] Gillingham, L. G. , S. Harrisjanz , and P. J. Jones . 2011. “Dietary Monounsaturated Fatty Acids Are Protective Against Metabolic Syndrome and Cardiovascular Disease Risk Factors.” Lipids 46: 209–228. 10.1007/s11745-010-3524-y. 21308420

[fsn371252-bib-0016] Gu, R. , L. Rybalov , A. Negrin , et al. 2019. “Metabolic Profiling of Different Parts of *Acer truncatum* From the Mongolian Plateau Using UPLC‐QTOF‐MS With Comparative Bioactivity Assays.” Journal of Agricultural and Food Chemistry 67: 1585–1597. 10.1021/acs.jafc.8b04035. 30675777

[fsn371252-bib-0017] He, Y. , S. Mao , Y. Zhao , and J. Yang . 2025. “Research Advances in the Synthesis, Metabolism, and Function of Chlorogenic Acid.” Food 14: 1914. 10.3390/foods14111914. PMC12154053 40509442

[fsn371252-bib-0018] Hills, M. J. 2004. “Control of Storage‐Product Synthesis in Seeds.” Current Opinion in Plant Biology 7: 302–308. 10.1016/j.pbi.2004.03.003. 15134751

[fsn371252-bib-0019] Hooper, L. , N. Martin , O. F. Jimoh , C. Kirk , E. Foster , and A. S. Abdelhamid . 2020. “Reduction in Saturated Fat Intake for Cardiovascular Disease.” Cochrane Database of Systematic Reviews 5: Cd011737. 10.1002/14651858.CD011737.pub2. 32428300 PMC7388853

[fsn371252-bib-0020] Huang, X. Z. , L. X. Tan , K. Gu , and C. Li . 2007. “Studies on Chemical Constituentsfrom Leaves of *Acer truncatum* .” Zhongguo Zhong Yao Za Zhi 32: 1544–1546. 17972585

[fsn371252-bib-0021] Hymowitz, T. , F. I. Collins , J. Panczner , and W. M. Walker . 1972. “Relationship Between the Content of Oil, Protein, and Sugar in Soybean Seed1.” Agronomy Journal 64: 613–616. 10.2134/agronj1972.00021962006400050019x.

[fsn371252-bib-0022] Ji, J. , X. Lu , H. Ma , et al. 2025. “Estimation of Plant Leaf Water Content Based on Spectroscopy.” Frontiers in Plant Science 16: 1609650. 10.3389/fpls.2025.1609650. 40530274 PMC12171197

[fsn371252-bib-0024] Li, G. Y. , L. I. Cong , O. L. Cheng , H. X. Zhong , Z. J. Cheng , and C. W. Xian . 2004. “Antioxidation Property of *Acer truncatum* Bunge Flavone Extract.” Chemical Researches 15: 42–44. 10.3969/j.issn.1008-1011.2004.02.013.

[fsn371252-bib-0026] Liang, Q. , W. Wang , F. Yuan , X. Liu , D. Li , and K. Q. Yang . 2019. “Characterization of *Yuanbaofeng* (*Acer truncatum* Bunge) Samaras: Oil, Fatty Acid, and Phytosterol Content.” Industrial Crops and Products 135: 344–351. 10.1016/j.indcrop.2019.04.032.

[fsn371252-bib-0027] Liu, H. , H. Li , J. Gu , et al. 2018. “Identification of the Candidate Proteins Related to Oleic Acid Accumulation During Peanut ( *Arachis hypogaea* L.) Seed Development Through Comparative Proteome Analysis.” International Journal of Molecular Sciences 19: 1235. 10.3390/ijms19041235. 29670063 PMC5979506

[fsn371252-bib-0028] Liu, J. , J. Wan , Y. Zhang , et al. 2023. “The Establishment of Comprehensive Quality Evaluation Model for Flavor Characteristics of Green Sichuan Pepper (*Zanthoxylum armatum* DC.) in Southwest China.” Food Chemistry: X 18: 100721. 10.1016/j.fochx.2023.100721. 37397205 PMC10314138

[fsn371252-bib-0029] Liu, P. , P. Wu , J. Bi , et al. 2024. “Development of an Analytic Method for Organosulfur Compounds in Welsh Onion and Its Use for Nutritional Quality Analysis of Five Typical Varieties in China.” Food Chemistry 441: 138237. 10.1016/j.foodchem.2023.138237. 38176137

[fsn371252-bib-0030] Liu, Y. , N. Shen , H. Xin , L. Yu , Q. Xu , and Y. Cui . 2023. “Unsaturated Fatty Acids in Natural Edible Resources, a Systematic Review of Classification, Resources, Biosynthesis, Biological Activities and Application.” Food Bioscience 53: 102790. 10.1016/j.fbio.2023.102790.

[fsn371252-bib-0031] Ma, M. , C. L. Hong , S. Q. An , and B. Li . 2003. “Seasonal, Spatial, and Interspecific Variation in Quercetin in *Apocynum venetum* and *Poacynum hendersonii*, Chinese Traditional Herbal Teas.” Journal of Agricultural and Food Chemistry 51: 2390–2393. 10.1021/jf021055i. 12670186

[fsn371252-bib-0032] Mais, E. , R. N. Alolga , S. L. Wang , L. O. Linus , X. Yin , and L. W. Qi . 2018. “A Comparative UPLC‐Q/TOF‐MS‐Based Metabolomics Approach for Distinguishing Zingiber Officinale Roscoe of Two Geographical Origins.” Food Chemistry 240: 239–244. 10.1016/j.foodchem.2017.07.106. 28946267

[fsn371252-bib-0033] Niggeweg, R. , A. J. Michael , and C. Martin . 2004. “Engineering Plants With Increased Levels of the Antioxidant Chlorogenic Acid.” Nature Biotechnology 22: 746–754. 10.1038/nbt966. 15107863

[fsn371252-bib-0034] Okubanjo, S. S. , S. M. Loveday , A. Ye , P. J. Wilde , and H. Singh . 2019. “Droplet‐Stabilized Oil‐In‐Water Emulsions Protect Unsaturated Lipids From Oxidation.” Journal of Agricultural and Food Chemistry 67: 2626–2636. 10.1021/acs.jafc.8b02871. 30608676

[fsn371252-bib-0035] Peng, J. , S. Zhu , X. Lin , et al. 2023. “Evaluation of Preharvest Melatonin on Soft Rot and Quality of Kiwifruit Based on Principal Component Analysis.” Food 12: 1414. 10.3390/foods12071414. PMC10093534 37048235

[fsn371252-bib-0036] Qiao, Q. , X. Wang , H. Ren , et al. 2019. “Oil Content and Nervonic Acid Content of *Acer truncatum* Seeds From 14 Regions in China.” Horticultural Plant Journal 5: 24–30. 10.1016/j.hpj.2018.11.001.

[fsn371252-bib-0037] Rawat, S. , A. K. Jugran , I. D. Bhatt , and R. S. Rawal . 2018. “Influence of the Growth Phenophases on the Phenolic Composition and Anti‐Oxidant Properties of *Roscoea Procera* Wall. In Western Himalaya.” Journal of Food Science and Technology 55: 578–585. 10.1007/s13197-017-2967-z. 29391622 PMC5785383

[fsn371252-bib-0038] Rodríguez‐Blázquez, S. , and E. Gómez‐Mejía . 2023. “Valorization of Prunus Seed Oils: Fatty Acids Composition and Oxidative Stability.” Molecules 28: 7045. 10.3390/molecules28207045. 37894525 PMC10609056

[fsn371252-bib-0039] Sambanthamurthi, R. , K. Sundram , and Y.‐A. Tan . 2000. “Chemistry and Biochemistry of Palm Oil.” Progress in Lipid Research 39: 507–558. 10.1016/S0163-7827(00)00015-1. 11106812

[fsn371252-bib-0040] Schwingshackl, L. , and G. Hoffmann . 2014. “Monounsaturated Fatty Acids, Olive Oil and Health Status: A Systematic Review and Meta‐Analysis of Cohort Studies.” Lipids in Health and Disease 13: 154. 10.1186/1476-511x-13-154. 25274026 PMC4198773

[fsn371252-bib-0023] Soyolt, K. , and S. Pei . 2001. “Wild Plants Used for the Folk Dietotherapy in Arhorchin Mongolians.” Zhong Yao Cai 24: 83–85. 10.3321/j.issn:1001-4454.2001.02.003. 11402735

[fsn371252-bib-0041] Tan, B. , Y. Wang , and X. Zhang . 2022. “Recent Studies on Protective Effects of Walnuts Against Neuroinflammation.” Nutrients 14: 4360. 10.3390/nu14204360. 36297047 PMC9609811

[fsn371252-bib-0042] Tohidi, B. , M. Rahimmalek , and A. Arzani . 2017. “Essential Oil Composition, Total Phenolic, Flavonoid Contents, and Antioxidant Activity of Thymus Species Collected From Different Regions of Iran.” Food Chemistry 220: 153–161. 10.1016/j.foodchem.2016.09.203. 27855883

[fsn371252-bib-0043] Vinyard, J. R. , G. S. Solis , M. L. Johnson , et al. 2025. “The Effects of Inclusion Level of an Extruded Flaxseed‐Pea Supplement on Nitrogen Balance and Flow of Amino and Fatty Acids in a Dual‐Flow Continuous Culture System.” Journal of Dairy Science 108: 12139–12147. 10.3168/jds.2025-26735. 40975275

[fsn371252-bib-0044] Wei, L. , G. Haiyan , C. Hangjun , W. Weijie , and F. Xiangjun . 2017. “Evaluation of Comprehensive Quality of Different Varieties of *Bayberry* Based on Principal Components Analysis (In Chinese With English Abstract).” Journal of Chinese Institute of Food Science and Technology 17: 161–171.

[fsn371252-bib-0045] Xu, Q. , J. Wang , D. Wang , et al. 2025. “Comprehensive Physicochemical Indicators Analysis and Quality Evaluation Model Construction for the Post‐Harvest Ripening *Rapeseeds* .” Food Chemistry 463: 141331. 10.1016/j.foodchem.2024.141331. 39305671

[fsn371252-bib-0046] Xu, Q. , J. Yu , D. Zhu , et al. 2022. “Nutritional Qua Lity Evaluation of Different Rice Varieties Based on Principal Component Analysis and Cluster Analysis (In Chinese With English Abstract).” China Rice 28: 1–8.

[fsn371252-bib-0047] Yeasmen, N. , and V. Orsat . 2023. “Phenolic Mapping and Associated Antioxidant Activities Through the Annual Growth Cycle of Sugar Maple Leaves.” Food Chemistry 428: 136882. 10.1016/j.foodchem.2023.136882. 37481905

[fsn371252-bib-0048] Yi, T. G. , Y. R. Yeoung , I. Y. Choi , and N. I. Park . 2019. “Transcriptome Analysis of *Asparagus officinalis* Reveals Genes Involved in the Biosynthesis of Rutin and Protodioscin.” PLoS One 14: e0219973. 10.1371/journal.pone.0219973. 31329616 PMC6645489

[fsn371252-bib-0049] Yildiz, A. Y. , S. Öztekin , and K. Anaya . 2025. “Effects of Plant‐Derived Antioxidants to the Oxidative Stability of Edible Oils Under Thermal and Storage Conditions: Benefits, Challenges and Sustainable Solutions.” Food Chemistry 479: 143752. 10.1016/j.foodchem.2025.143752. 40086382

[fsn371252-bib-0050] Yin, Y. , C. Sui , F. Meng , P. Ma , and Y. Jiang . 2017. “The Omega‐3 Polyunsaturated Fatty Acid Docosahexaenoic Acid Inhibits Proliferation and Progression of Non‐Small Cell Lung Cancer Cells Through the Reactive Oxygen Species‐Mediated Inactivation of the PI3K/Akt Pathway.” Lipids in Health and Disease 16: 87. 10.1186/s12944-017-0474-x. 28468627 PMC5415787

[fsn371252-bib-0051] Yuan, S. N. , M. X. Wang , J. L. Han , et al. 2023. “Improved Colonic Inflammation by Nervonic Acid via Inhibition of NF‐kappaB Signaling Pathway of DSS‐Induced Colitis Mice.” Phytomedicine: International Journal of Phytotherapy and Phytopharmacology 112: 154702. 10.1016/j.phymed.2023.154702. 36764096

[fsn371252-bib-0052] Zhang, F. , S. Y. Luo , Y. B. Ye , et al. 2008. “The Antibacterial Efficacy of an Aceraceous Plant [Shantung Maple (*Acer truncatum* Bunge)] May Be Related to Inhibition of Bacterial Beta‐Oxoacyl‐Acyl Carrier Protein Reductase (FabG).” Biotechnology and Applied Biochemistry 51: 73–78. 10.1042/ba20070255. 18208374

[fsn371252-bib-0053] Zhang, Y. , H. Gong , X. Cui , et al. 2023. “Integrated Lipidomic and Transcriptomic Analyses Reveal the Mechanism of Lipid Biosynthesis and Accumulation During Seed Development in Sesame.” Frontiers in Plant Science 14: 1211040. 10.3389/fpls.2023.1211040. 37426956 PMC10325577

[fsn371252-bib-0055] Zhao, W. H. , L. F. Gao , W. Gao , et al. 2011. “Weight‐Reducing Effect of *Acer truncatum* Bunge May Be Related to the Inhibition of Fatty Acid Synthase.” Natural Product Research 25: 422–431. 10.1080/14786419.2010.488625. 21328136

[fsn371252-bib-0056] Zhao, W. H. , J. F. Zhang , W. Zhe , Y. X. Zhang , and W. X. Tian . 2006. “The Extract of Leaves of *Acer truncatum* Bunge: A Natural Inhibitor of Fatty Acid Synthase With Antitumor Activity.” Journal of Enzyme Inhibition and Medicinal Chemistry 21: 589–596. 10.1080/14756360600774579. 17194032

[fsn371252-bib-0057] Zhao, X. , X. Xiang , J. Huang , Y. Ma , J. Sun , and D. Zhu . 2021. “Studying the Evaluation Model of the Nutritional Quality of Edible Vegetable Oil Based on Dietary Nutrient Reference Intake.” ACS Omega 6: 6691–6698. 10.1021/acsomega.0c05544. 33748582 PMC7970471

[fsn371252-bib-0058] Zhou, T. , Q. Xing , J. Bu , W. Han , and Z. Shen . 2024. “Integrated Metabolomic and Transcriptomic Analysis Reveals the Regulatory Mechanisms of Flavonoid and Alkaloid Biosynthesis in the New and Old Leaves of *Murraya tetramera huang* .” BMC Plant Biology 24: 499. 10.1186/s12870-024-05066-9. 38840069 PMC11151518

[fsn371252-bib-0059] Zhu, X. , G. Ding , S. Ren , J. Xi , and K. Liu . 2024. “The Bioavailability, Absorption, Metabolism, and Regulation of Glucolipid Metabolism Disorders by Quercetin and Its Important Glycosides: A Review.” Food Chemistry 458: 140262. 10.1016/j.foodchem.2024.140262. 38944925

